# Visually Entrained Theta Oscillations Increase for Unexpected Events in the Infant Brain

**DOI:** 10.1177/0956797619876260

**Published:** 2019-10-11

**Authors:** Moritz Köster, Miriam Langeloh, Stefanie Hoehl

**Affiliations:** 1 Institute of Psychology, Free University Berlin; 2 Max Planck Institute for Human Cognitive and Brain Sciences, Leipzig, Germany; 3 Department of Psychology, Heidelberg University; 4 Faculty of Psychology, University of Vienna

**Keywords:** infant cognition, rhythmic visual brain stimulation, steady-state visually evoked potentials, violation of expectations, theta and alpha neural oscillations, open data, open materials, preregistered

## Abstract

Infants form basic expectations about their physical and social environment, as indicated by their attention toward events that violate their expectations. Yet little is known about the neuronal processing of unexpected events in the infant brain. Here, we used rhythmic visual brain stimulation in 9-month-olds (*N* = 38) to elicit oscillations of the theta (4 Hz) and the alpha (6 Hz) rhythms while presenting events with unexpected or expected outcomes. We found that visually entrained theta oscillations sharply increased for unexpected outcomes, in contrast to expected outcomes, in the scalp-recorded electroencephalogram. Visually entrained alpha oscillations did not differ between conditions. The processing of unexpected events at the theta rhythm may reflect learning processes such as the refinement of infants’ basic representations. Visual brain-stimulation techniques provide new ways to investigate the functional relevance of neuronal oscillatory dynamics in early brain development.

In their first year after birth, infants develop basic concepts about their physical and social environment (Spelke & Kinzler, 2007), as indicated by their responses to unexpected events. For example, infants show basic expectations about physical laws (Spelke, Breinlinger, Macomber, & Jacobsen, 1992), numbers (Wynn, 1992), and human actions (Reid et al., 2009). Differential looking times and event-related responses in an electroencephalogram (EEG) to unexpected events are commonly interpreted as an increase in infants’ attention. In addition, recent evidence suggests that infants take unexpected events as an opportunity to learn (Stahl & Feigenson, 2015). In the present study, we use a rhythmic visual-stimulation approach to scrutinize the neuronal dynamics involved in infants’ processing of unexpected events.

Neuronal oscillations reflect the synchronization of activity within and across nerve-cell populations (Fries, 2015). Specifically, different brain rhythms are associated with distinct cognitive processes. The processing of novel information is accompanied by increases in theta oscillations (3–8 Hz) and decreases in alpha oscillations (8–14 Hz) in adults (Friese et al., 2013) and children (Köster, Haese, & Czernochowski, 2017). Whereas alpha suppression marks visual attention processes (Jensen & Mazaheri, 2010), theta oscillations are involved in learning processes (Köster, Finger, Graetz, Kater, & Gruber, 2018). In the infant brain, alpha suppression has also been associated with attention (Hoehl, Michel, Reid, Parise, & Striano, 2014), and increased theta oscillation has been related to learning (Begus, Southgate, & Gliga, 2015) and to the processing of unexpected events (Berger, Tzur, & Posner, 2006).

Neuronal oscillatory dynamics can be experimentally manipulated by rhythmic perceptual-stimulation techniques. For example, visually flickering stimuli elicit neuronal oscillations in a specific stimulation frequency (steady-state visually evoked potentials, or SSVEPs; Müller, Malinowski, Gruber, & Hillyard, 2003). In adults, entrained theta oscillations enhance subsequent memory performance compared with entrained alpha oscillations (Köster, Martens, & Gruber, 2019) or delta oscillations (~2 Hz; Clouter, Shapiro, & Hanslmayr, 2017). Testing the resonating network dynamics of different stimulation conditions is a critical addition to the measurement of ongoing oscillatory dynamics, since such testing allows for an experimental manipulation and functional dissociation among different neuronal frequencies (Herrmann, Struber, Helfrich, & Engel, 2016).

In the present study, we applied rhythmic visual brain stimulation in 9-month-olds. To test the involvement of the theta and alpha rhythms in the processing of unexpected events, we visually flickered the events at 4 Hz and 6 Hz—corresponding to infants’ theta and alpha rhythms—and recorded their scalp EEG. For unexpected events, we expected a decrease in alpha (indexing attention processes) and an increase in theta (indexing learning processes; see the preregistration at https://aspredicted.org/ve2qn.pdf).

## Method

### Subjects

The final sample consisted of 38 nine-month-old infants (14 girls; age: *M* = 9.4 months, *SD* = 7 days). Previous visual EEG studies used final samples of between 10 and 50 subjects (e.g., Hoehl et al., 2014; Reid et al., 2009), but because we planned to apply a new EEG paradigm, effect sizes could not be estimated. Consequently, we aimed for a final sample of 35 infants, which is toward the upper end of previous sample-size ranges. Thirteen additional infants were tested but excluded from the final sample because of failure to provide the minimum number of 20 artifact-free trials (*n* = 9) or because of fussiness (*n* = 4). This attrition rate is rather low for infant visual EEG studies. We selected this age group because previous studies indicated that infants demonstrate violation-of-expectation responses for the knowledge domains of interest by the age of 9 months or even earlier (Reid et al., 2009; Spelke et al., 1992). Subjects were healthy full-term infants from a midsized German city. Informed written consent was obtained from each subject’s parent before the experiment, and the experimental procedure was approved by the local ethics committee. This study was preregistered at AsPredicted.org (https://aspredicted.org/ve2qn.pdf).

### Infants’ theta and alpha frequency

To get an impression of 9-month-olds’ theta and alpha frequencies for violation-of-expectation stimuli, we visually explored the theta and alpha power in existing data before conducting the present study. Using an independent sample of 9-month-olds, Reid and colleagues (2009) showed infants the same action stimuli that we used in the present study; for details on the experimental procedures, see Reid et al. (2009). Specifically, we looked for an increase in theta and a decrease in alpha within the range of 3 Hz to 10 Hz on stimulus onset. Our visual exploration was consistent with previous literature, showing an increase of around 4 Hz for theta (Begus et al., 2015) and a decrease of around 6 Hz for alpha (Hoehl et al., 2014) in infants around 9 months of age. Thus, in the present study, we used 4 Hz for the stimulation of infants’ theta rhythm and 6 Hz for the stimulation of infants’ alpha rhythm.

### Stimuli and procedure

**Fig. 1.**
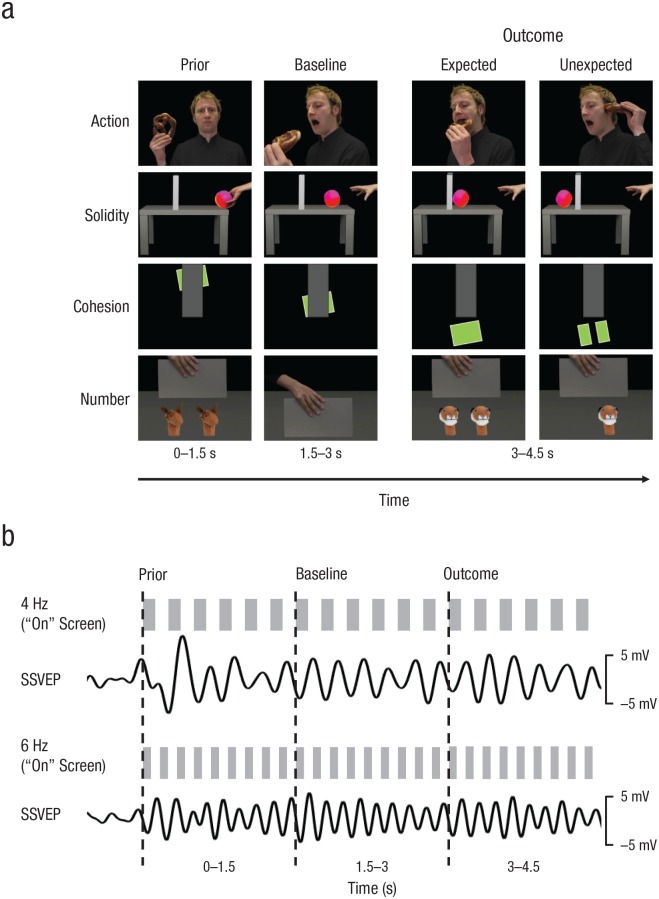
Example trial sequences and rhythmic visual stimulation. In each trial (a), infants saw stimuli from one of four different core knowledge domains (action, solidity, cohesion, or number). The first two pictures represented a physical or social event (prior and baseline pictures), and the third picture represented the outcome of that event, which could be expected or unexpected. Each picture was presented for 1.5 s. The schematic (b) illustrates the rhythmic stimulation protocol and the resulting neuronal oscillatory dynamics (steady-state visually evoked potentials, or SSVEPs). The picture sequences were visually flickered at a theta (4-Hz) or an alpha (6-Hz) frequency using the “on” and “off” screens of a 60-Hz CRT monitor. In the illustration, gray bars indicate “on” (the stimulus on screen) and “off” (a black screen; see also Videos S1 to S8). The visual stimulation elicited a clear SSVEP response, here averaged over occipital channels (O1, Oz, O2) and all stimuli presented in the specific frequency (filtered at ±1 Hz around the driving frequency).

Stimuli were based on four classical violation-of-expectation paradigms for the core knowledge domains—*action, number, solidity*, and *cohesion* (Reid et al., 2009; Spelke et al., 1992; Wynn, 1992)—in four variations each (see Fig. 1; see Fig. S1 in the Supplemental Material available online for the complete stimulus set). Each stimulus trial consisted of three static images that, when presented in succession, depicted a scenario with a clearly expectable outcome.

In a within-subjects design, each of the 16 sequences was presented four times, one time in each condition, at each of the two stimulation frequencies (4-Hz theta or 6-Hz alpha) combined with each outcome (expected or unexpected). This resulted in a total of 64 distinct trials presented in 16 blocks. In each block, four variations of a single knowledge domain (action, number, solidity, or cohesion) were presented in one of the two frequencies (theta or alpha) and with one of the two outcomes (unexpected or expected). The order of the core knowledge domains, frequencies, and outcomes was counterbalanced across infants. Furthermore, we counterbalanced the order of the four stimulus domains.

Every trial began with an attention grabber (a yellow duck with sound for 1 s), followed by a black screen (variable duration of 0.5–0.75 s) and the three stimulus pictures (4.5 s). The first two pictures showed the initiation of an event or action (0–3 s, or 1.5 s for each picture; see Fig. 1a), followed by the picture presenting the expected or the unexpected outcome (1.5 s; see Fig. 1a). Stimuli for sequences with expected versus unexpected outcomes were counterbalanced for the cohesion and the number stimuli (i.e., the unexpected outcome could be connected or unconnected objects as well as one or two objects) and were matched in terms of luminance and contrast for action and solidity (all *p*s > .30). Stimuli were presented via the Psychophysics Toolbox (Version 0.20170103; Brainard, 1997) in MATLAB (Version 9.1; The MathWorks, Natick, MA).

Infants sat on their parent’s lap at a viewing distance of about 80 cm from the stimulus monitor. Sequences were presented at the center of a 17-in. CRT screen at a visual angle of approximately 15.0° × 15.0° for the focal event (see Fig. S2 in the Supplemental Material). We presented infants with 64 trials. If infants were still attentive after the first presentation of 64 trials, the presentation was restarted a second time. The session ended when the infant no longer attended to the screen. Infants were video-recorded throughout the experiment for off-line coding of gaze behavior (see below).

### Visual brain entrainment

We visually entrained a 4-Hz theta rhythm and a 6-Hz alpha rhythm by controlling the presentation at every refresh cycle of a 60-Hz CRT monitor (one refresh cycle = 16.67 ms). The flickering was established by showing either the stimulus (“on”) or a black screen (“off”). To establish a flicker rate of 6 Hz, we presented the stimulus at a duty cycle of 5:5 (i.e., by alternating five “on” and five “off” frames at a refresh rate of 60 Hz). For 4 Hz, we used combined duty cycles of 7:8 and 8:7 “on” and “off” frames, respectively, to ensure an equal number of “on” and “off” screens between both conditions (4 Hz and 6 Hz). See Figure 1b for an illustration of the stimulation protocol and the resulting SSVEP at occipital recording sites.

### Gaze behavior

A video recording of the infants was used to monitor their looking time. Gaze behavior was coded off-line on the basis of the video recordings; agreement between two independent raters who analyzed more than 25% of the trials was excellent (intraclass correlation coefficient = .979).

### Electroencephalogram

#### Apparatus

The EEG was recorded continuously in a shielded room using 30 Ag/AgCl ring electrodes from 30 scalp locations determined according to the international 10-20 system. Data were recorded with a Twente Medical Systems (Oldenzaal, The Netherlands) 32-channel Refa amplifier at a sampling rate of 500 Hz. Horizontal and vertical electrooculograms were recorded bipolarly. Impedances were controlled at the beginning of the experiment and accepted when below 10 kΩ. Electrode signals were referenced to the vertex (Cz).

#### Preprocessing

EEG data were preprocessed and analyzed in MATLAB (Version R2017b). EEG signals were band-pass filtered from 1 Hz to 120 Hz and segmented into epochs from −1.5 s to 6 s with respect to the stimulus onset. Trials in which infants did not watch the complete 4.5-s sequence were excluded from the analyses. Furthermore, noisy trials were identified visually and discarded (~10% of all trials), and noisy electrodes were interpolated on the basis of spherical information. Eyeblinks and muscle artifacts were detected using an independent component procedure (ICA) and removed after visual inspection by the first author. To avoid any bias, we identified and removed the ICAs across the whole data set, including all experimental conditions—both frequencies, both outcome conditions, all stimulus categories. We first removed the noisiest channels and trials (on the basis of visual inspection) and then conducted the ICA artifact rejection. If necessary, we repeated this procedure. A minimum of 20 artifact-free trials across conditions was required for an infant to be included in the statistical analyses. Infants in the final sample contributed 20 to 65 trials (*M* = 35.8, *SD* = 11.4), with no significant differences in the number of trials between frequencies and conditions (all *p*s > .371).

#### Steady-state visually evoked potentials

**Fig. 2.**
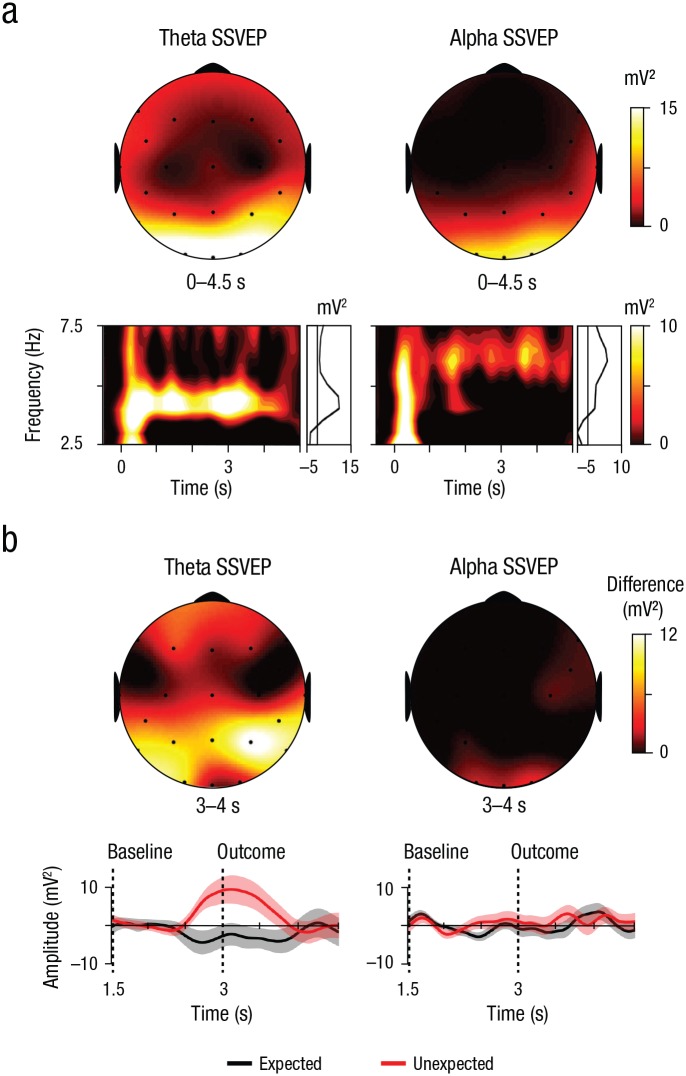
Steady-state visually evoked potential (SSVEP) responses to visual rhythmical stimulation. In (a), the topographic maps display the SSVEPs at the entrained theta and alpha frequency (0–4.5 s, baseline from −0.5 s to −0.25 s) collapsed across both outcome conditions (expected, unexpected). The time-frequency heat maps display the spectral power at posterior electrodes (O1, Oz, O2, P3, P4, Pz, P7, P8) over time, and the line plots to the right of the heat maps show the averaged spectral power (0−4.5 s). In (b), the topographic maps display the difference in SSVEP power between the unexpected and the expected conditions in response to the outcome (3–4 s) and in contrast to the presentation of the second picture, which was used as a baseline (1.5–2.5 s). Maps are shown separately for the theta and alpha frequencies. The outcome picture (expected vs. unexpected) was presented at the 3-s point during each trial. The line plots display the spectral power at posterior electrodes for each outcome. Error bands correspond to ±1 *SE*.

The main analyses focused on the SSVEPs elicited by theta and alpha stimulation at the posterior electrodes (O1, Oz, O2, P3, P4, Pz, P7, P8), given the topography of the SSVEP signals (see Fig. 2a). Specifically, the evoked spectral power was obtained by complex Morlet’s wavelets (Morlet parameter *m* = 7) at a resolution of 0.5 Hz for theta and alpha for each channel individually (Formulas 1 to 4 in Kaspar, Hassler, Martens, Trujillo-Barreto, & Gruber, 2010). We identified the peak SSVEP signals as between 3 and 5 Hz for theta oscillations (*M* = 4.10, *SD* = 0.50) and between 5 and 7 Hz for alpha oscillations (*M* = 5.90, *SD* = 0.70). Specifically, we defined the frequency with the highest spectral increase across all occipital and parietal electrodes (O1, Oz, O2, P3, P4, Pz, P7, P8) across the whole time window (0–4.5 s; baseline ranged from −0.5 s to −0.25 s before stimulus onset) and across all trials of the specific stimulation condition (4 Hz or 6 Hz, respectively). Note that individual frequency adjustment was critical in the present study because for some infants, the endogenous response was just above or below the external stimulation frequency (see Fig. S3 in the Supplemental Material). Also note that the same individual frequency was used to test the theta response in the alpha-stimulation condition (see the control analysis in the last paragraph of the Results section). Because the external stimulation highly disrupts the naturally occurring frequency spectrum (see Fig. 1a), the theta peak in the theta-stimulation condition is the best estimate for an individual theta frequency in the present data.

The grand mean SSVEP signal was quantified by subtracting a prestimulus baseline (from −0.5 s to −0.25 s) from the SSVEP power. The main analysis focused on the difference in SSVEP power for a 1-s time window after the onset of the outcome stimulus (3–4 s), in contrast to the 1-s presentation of the second picture of the sequence (1.5–2.5 s), subtracted as a baseline. Subsequently, we averaged signal power at individual frequencies across occipital and parietal electrodes (O1, Oz, O2, P3, P4, Pz, P7, P8) for all statistical comparisons.

The results differed slightly from our original prediction (see the preregistration at https://aspredicted.org/ve2qn.pdf). First, the SSVEP signal and difference of interest spread into parietal cortical networks. Thus, we included all posterior electrodes in the analysis (O1, Oz, O2, P3, P4, Pz, P7, P8; note that this resembles closely the condition difference at parietal electrodes in some previous SSVEP studies; e.g., Köster et al., 2019). Second, because of the edge artifact of the SSVEP (2.5–3 s) and the temporal characteristics of the SSVEP effects—the difference of interest lasted about 1 s—we used 1.5 s to 2.5 s as a baseline and 3 s to 4 s for the main analyses, instead of using the whole presentation durations of the baseline and the outcome picture. We did not include stimulus category as an additional factor in the analysis of variance (ANOVA), because not enough infants provided sufficient trials for each cell of this ANOVA. Note that the actual effects were very close to what we predicted, given that this is the first visual-entrainment study testing infants’ violation-of-expectation responses.

## Results

Visual brain entrainment elicited clear SSVEPs at posterior recording sites (Fig. 2a). Throughout the stimulus presentation (0–4.5 s), theta and alpha SSVEPs were significantly above the prestimulus baseline level, both *t*s(37) > 7.46, *p* < .001, with higher SSVEP power for entrained theta compared with alpha, *t*(37) = 3.38, *p* = .002.

To test for the effect of unexpected outcomes, we entered the posterior SSVEP for the outcome stimulus (3 s–4 s) into a Frequency (theta, alpha) × Outcome (expected, unexpected) repeated measures ANOVA. We found a significant main effect of outcome, *F*(1, 37) = 6.72, *p* = .014, η_*p*_^2^ = .154, but no main effect of frequency, *F*(1, 37) = 0.03, *p* = .858, η_*p*_^2^ = .001. Furthermore, there was a trend for a Frequency × Outcome interaction, *F*(1, 37) = 3.37, *p* = .075, η_*p*_^2^ = .083, in the expected direction (one-sided *p* = .037; see the preregistration at https://aspredicted.org/ve2qn.pdf). Post hoc *t* tests revealed a sharp increase in SSVEP power for the onset of unexpected compared with expected outcomes for the theta stimulation, *t*(37) = 2.85, *p* = .007, but no difference was found for the alpha stimulation, *t*(37) = 0.41, *p* = .684 (Fig. 2b).

To test the possibility that the differential response to expected versus unexpected outcomes during the theta 4-Hz presentation reflected a mere endogenous effect independent from the 4-Hz stimulation, we conducted a control analysis, in which we tested the difference in the theta rhythm for the processing of expected versus unexpected events during the alpha 6-Hz stimulation. The theta frequency during the alpha stimulation did not differ between the expected and unexpected conditions in the first second of the outcome picture (3–4 s), *t*(37) = 0.62, *p* = .536.

## Discussion

Visually entrained theta oscillations increased for unexpected events, which violated infants’ basic expectations. The theta rhythm is a phylogenetically preserved mechanism for neuronal organization in the mammalian brain (Lisman & Jensen, 2013) with a specific role for learning in human adults (Friese et al., 2013; Köster et al., 2018), children (Köster et al., 2017), and infants (Begus et al., 2015). Thus, increased theta SSVEPs for unexpected events may reflect learning processes—that is, the integration of novel information to refine basic expectations. This idea fits neatly into the predictive-processing framework, which proposes the minimization of prediction errors as a general working principle of the brain (Friston, 2011); it also fits with the finding that infants try to make sense of unexpected outcomes by increased exploration behavior following violation-of-expectation experiences (Stahl & Feigenson, 2015).

Entrained alpha oscillations, which are associated with attention, did not differ between outcomes (Clouter et al., 2017; Köster et al., 2019), although we expected a decrease in entrained alpha oscillations for unexpected events. The suppression in the alpha SSVEP, as opposed to an increase, has been associated with attention processes in previous research (Gulbinaite, Roozendaal, & VanRullen, 2019).

The strong impact of visual entrainment on infants’ neuronal dynamics (see Figs. 1b and 2a) underlines that we manipulated rhythmic neural processes beyond a mere amplification of ongoing neuronal signals. In future research, it would be intriguing to further substantiate the specificity of the theta rhythm in the processing of unexpected events and the causal role of the theta rhythm in encoding processes. The theta rhythm may be contrasted to further stimulation frequencies (Clouter et al., 2017), as in the adult literature, and compared with behavioral-learning outcomes (Köster et al., 2019), such as infants’ exploration behavior (Stahl & Feigenson, 2015).

To summarize, we established visual brain entrainment in the maturing infant brain, which elicited robust SSVEP responses and revealed a frequency-specific dissociation between expected and unexpected events. Thus, the present study is a critical first step and will open up new pathways for the experimental manipulation and investigation of the neuronal oscillatory dynamics underlying cognitive processes in infancy—a period of intense brain development and learning.

## Supplemental Material

Langeloh_OpenPracticesDisclosure_rev2 – Supplemental material for Visually Entrained Theta Oscillations Increase for Unexpected Events in the Infant Brain Click here for additional data file. Supplemental material, Langeloh_OpenPracticesDisclosure_rev2 for Visually Entrained Theta Oscillations Increase for Unexpected Events in the Infant Brain by Moritz Köster, Miriam Langeloh and Stefanie Hoehl in Psychological Science

## Supplemental Material

Langeloh_Supplemental_Figures_and_Video_Captions – Supplemental material for Visually Entrained Theta Oscillations Increase for Unexpected Events in the Infant Brain Click here for additional data file. Supplemental material, Langeloh_Supplemental_Figures_and_Video_Captions for Visually Entrained Theta Oscillations Increase for Unexpected Events in the Infant Brain by Moritz Köster, Miriam Langeloh and Stefanie Hoehl in Psychological Science

## References

[bibr1-0956797619876260] Begus K. Southgate V. Gliga T. (2015). Neural mechanisms of infant learning: Differences in frontal theta activity during object exploration modulate subsequent object recognition. Biology Letters, 11(5), Article 20150041. doi:10.1098/rsbl.2015.0041 PMC4455734 26018832

[bibr2-0956797619876260] Berger A. Tzur G. Posner M. I. (2006). Infant brains detect arithmetic errors. Proceedings of the National Academy of Sciences, USA, 103, 12649–12653. doi:10.1073/pnas.0605350103 PMC1567933 16894149

[bibr3-0956797619876260] Brainard D. H (1997). The Psychophysics Toolbox. Spatial Vision, 10, 433–436. 9176952

[bibr4-0956797619876260] Clouter A. Shapiro K. L. Hanslmayr S. (2017). Theta phase synchronization is the glue that binds human associative memory. Current Biology, 27, 3143–3148. doi:10.1016/j.cub.2017.09.001 28988860

[bibr5-0956797619876260] Fries P. (2015). Rhythms for cognition: Communication through coherence. Neuron, 88, 220–235. doi:10.1016/j.neuron.2015.09.034 26447583 PMC4605134

[bibr6-0956797619876260] Friese U. Köster M. Hassler U. Martens U. Trujillo-Barreto N. Gruber T. (2013). Successful memory encoding is associated with increased cross-frequency coupling between frontal theta and posterior gamma oscillations in human scalp-recorded EEG. NeuroImage, 66, 642–647. doi:10.1016/j.neuroimage.2012.11.002 23142278

[bibr7-0956797619876260] Friston K. (2011). What is optimal about motor control? Neuron, 72, 488–498. doi:10.1016/j.neuron.2011.10.018 22078508

[bibr8-0956797619876260] Gulbinaite R. Roozendaal D. H. M. VanRullen R. (2019). Attention differentially modulates the amplitude of resonance frequencies in the visual cortex. NeuroImage, 203, Article 116146. doi:10.1016/j.neuroimage.2019.116146 31493535

[bibr9-0956797619876260] Herrmann C. S. Struber D. Helfrich R. F. Engel A. K. (2016). EEG oscillations: From correlation to causality. International Journal of Psychophysiology, 103, 12–21. doi:10.1016/j.ijpsycho.2015.02.003 25659527

[bibr10-0956797619876260] Hoehl S. Michel C. Reid V. M. Parise E. Striano T. (2014). Eye contact during live social interaction modulates infants’ oscillatory brain activity. Social Neuroscience, 9, 300–308. doi:10.1080/17470919.2014.884982 24506533

[bibr11-0956797619876260] Jensen O. Mazaheri A. (2010). Shaping functional architecture by oscillatory alpha activity: Gating by inhibition. Frontiers in Human Neuroscience, 4, Article 186. doi:10.3389/fnhum.2010.00186 PMC2990626 21119777

[bibr12-0956797619876260] Kaspar K. Hassler U. Martens U. Trujillo-Barreto N. Gruber T. (2010). Steady-state visually evoked potential correlates of object recognition. Brain Research, 1343, 112–121. doi:10.1016/j.brainres.2010.04.072 20450897

[bibr13-0956797619876260] Köster M. Finger H. Graetz S. Kater M. Gruber T. (2018). Theta-gamma coupling binds visual perceptual features in an associative memory task. Scientific Reports, 8(1), Article 17688. doi:10.1038/s41598-018-35812-7 PMC6283876 30523336

[bibr14-0956797619876260] Köster M. Haese A. Czernochowski D. (2017). Neuronal oscillations reveal the processes underlying intentional compared to incidental learning in children and young adults. PLOS ONE, 12(8), Article e0182540. doi:10.1371/journal.pone.0182540 PMC5540547 28767720

[bibr15-0956797619876260] Köster M. Martens U. Gruber T. (2019). Memory entrainment by visually evoked theta-gamma coupling. NeuroImage, 188, 181–187. doi:10.1016/j.neuroimage.2018.12.002 30529173

[bibr16-0956797619876260] Lisman J. E. Jensen O. (2013). The theta-gamma neural code. Neuron, 77, 1002–1016. doi:10.1016/j.neuron.2013.03.007 23522038 PMC3648857

[bibr17-0956797619876260] Müller M. Malinowski P. Gruber T. Hillyard S. A. (2003). Sustained division of the attentional spotlight. Nature, 424, 309–312. doi:10.1038/nature01812 12867981

[bibr18-0956797619876260] Reid V. M. Hoehl S. Grigutsch M. Groendahl A. Parise E. Striano T. (2009). The neural correlates of infant and adult goal prediction: Evidence for semantic processing systems. Developmental Psychology, 45, 620–629. doi:10.1037/a0015209 19413420

[bibr19-0956797619876260] Spelke E. S. Breinlinger K. Macomber J. Jacobsen K. (1992). Origins of knowledge. Psychological Review, 99, 605–632. doi:10.1037/0033-295X.99.4.605 1454901

[bibr20-0956797619876260] Spelke E. S. Kinzler K. D. (2007). Core knowledge. Developmental Science, 10, 89–96. doi:10.1111/j.1467-7687.2007.00569.x 17181705

[bibr21-0956797619876260] Stahl A. E. Feigenson L. (2015). Observing the unexpected enhances infants’ learning and exploration. Science, 348, 91–94. doi:10.1126/science.aaa3799 25838378 PMC5861377

[bibr22-0956797619876260] Wynn K. (1992). Addition and subtraction by human infants. Nature, 358, 749–750. doi:10.1038/358749a0 1508269

