# Supplementary material for: Visually Entrained Theta Oscillations Increase for Unexpected Events in the Infant Brain
Source: Psychol Sci. 2019 Oct 11;30(11):1656–63. doi: 10.1177/0956797619876260 (PMC6843601; doi:10.1177/0956797619876260)
Supplement: Langeloh_Supplemental_Figures_and_Video_Captions – Supplemental material for Visually Entrained Theta Oscillations Increase for Unexpected Events in the Infant Brain [file Langeloh_Supplemental_Figures_and_Video_Captions.pdf]

Supplemental Online Material

Visually entrained theta oscillations increase for unexpected events in the infant brain

Moritz Köster*, Miriam Langeloh*, & Stefanie Hoehl

*MK and ML contributed equally to this work

A

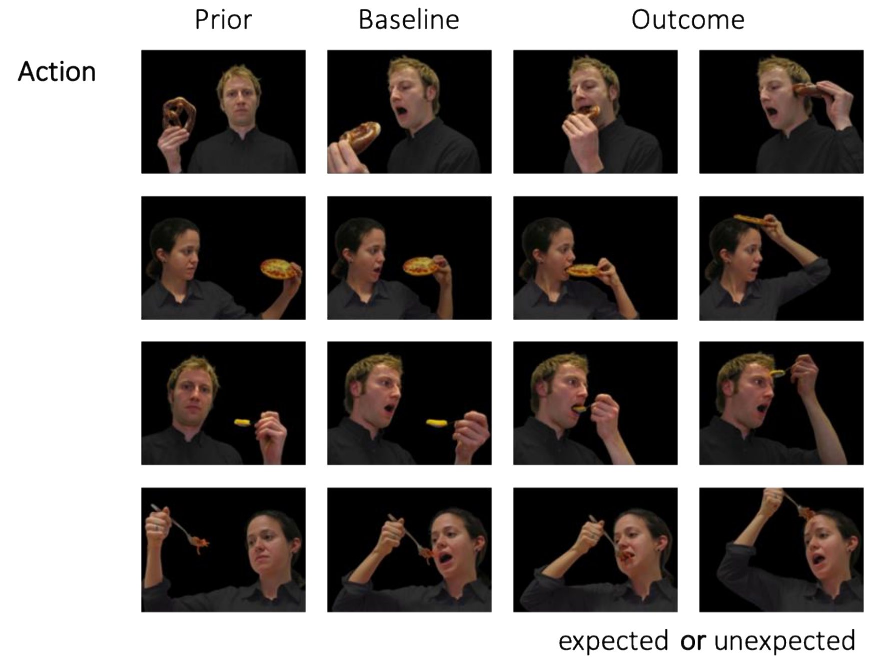
Prior
Baseline
Outcome
Action
expected or unexpected

B

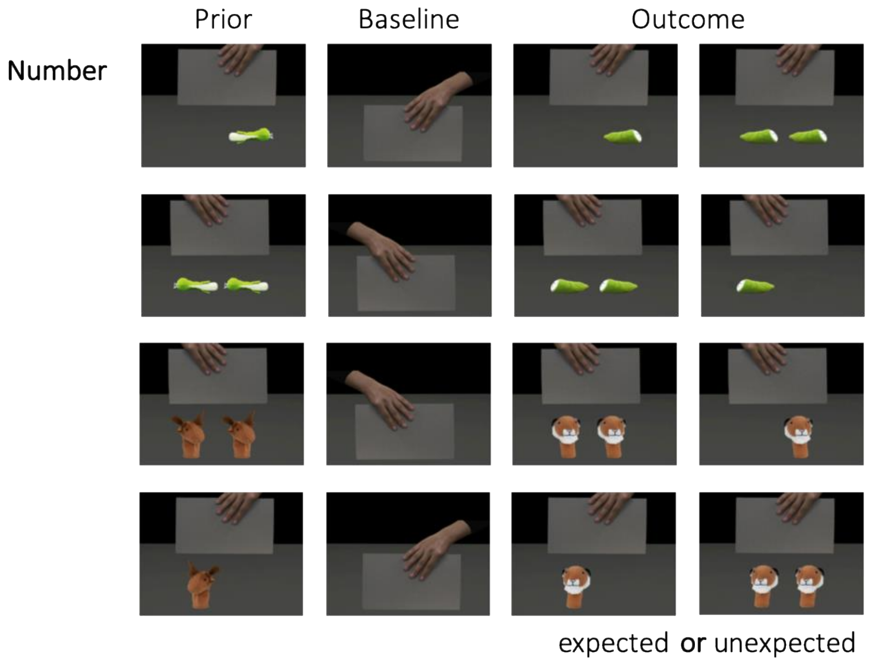
Prior
Baseline
Outcome
Number
expected or unexpected

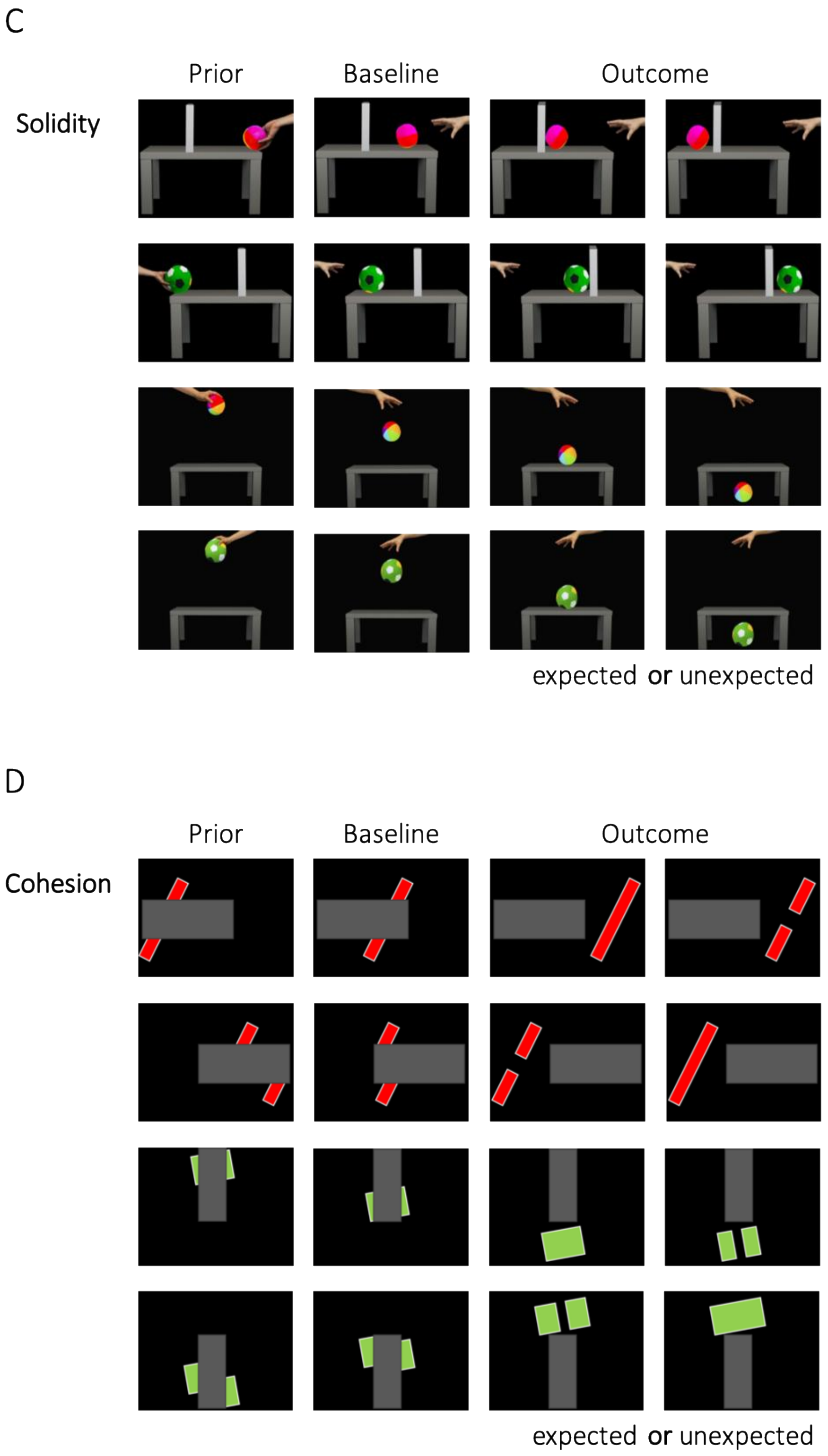

Fig. S1. Stimulus sets. Complete stimulus sets used in the (A) action, (B) number, (C) solidity and (D) cohesion domain. Each trial consisted of three stimulus pictures. The first two pictures showed the initiation of an event or action (prior and baseline picture) followed by the third picture, presenting either an expected or an unexpected outcome.

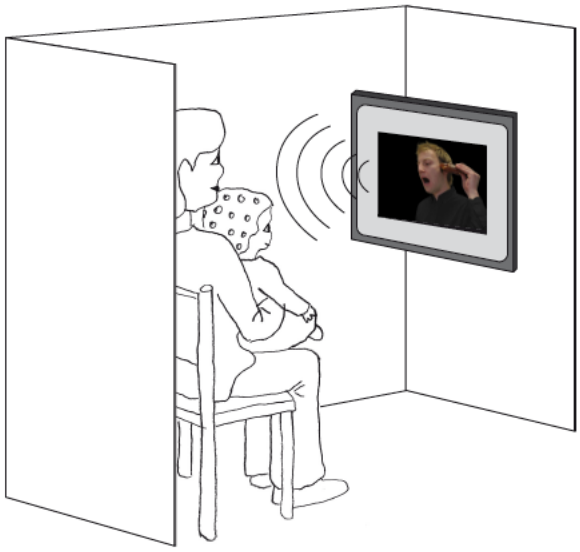

Fig. S2. Illustration of the infancy EEG set-up. During stimulus presentation infants sat on their parent’s lab in a sound-attenuated electromagnetically-shielded room. Stimuli (Fig. S1) were flickered in infants’ theta (4 Hz) and alpha frequency (6 Hz) on a 17-inch 60 Hz CRT monitor.

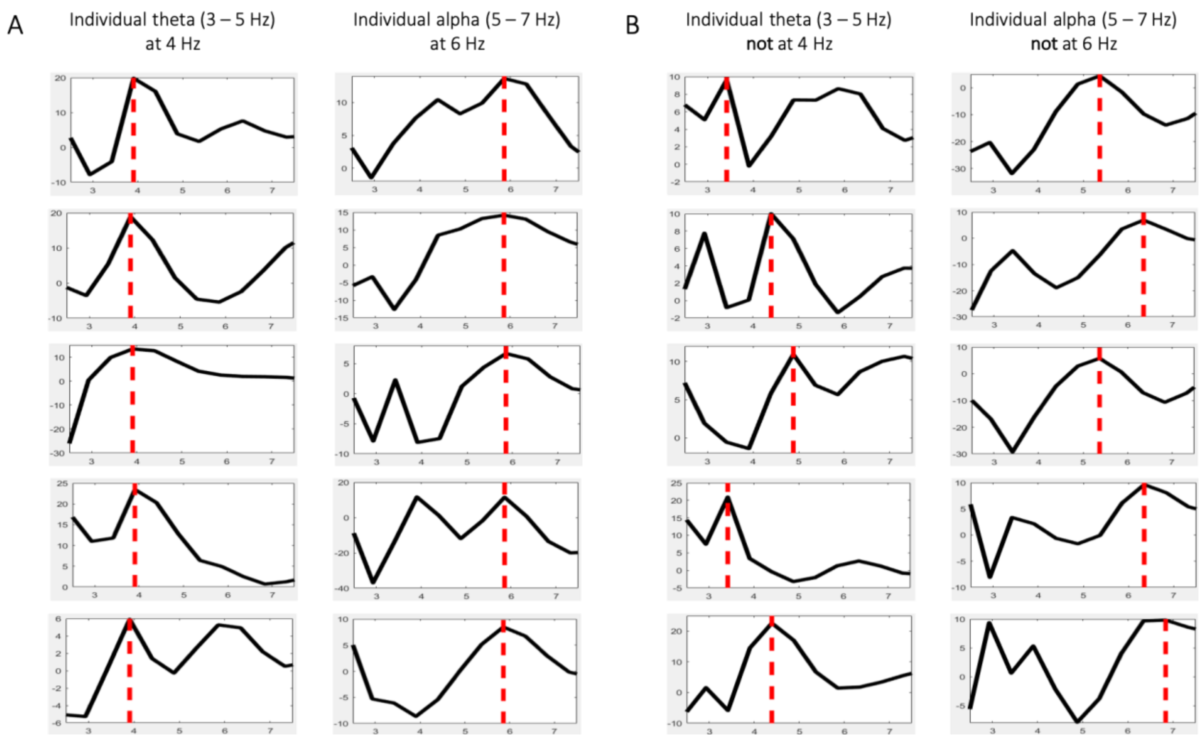

Fig. S3. Exemplary individual spectral plots during 4 Hz and 6 Hz stimulation. Power (mV^2) is plotted on the y-axis and frequency (Hz) is plotted on the x-axis. Power was calculated across the whole stimulus presentation (0 – 4.5 s). Red dashed lines indicate individual theta and alpha peaks during the respective stimulation frequency. A) Illustration of 5 participants, in which the entrained oscillatory dynamics were exactly at the stimulation frequency for 4 Hz and 6 Hz, respectively. B) Illustration of 5 participants, in which the entrained oscillatory dynamics were slightly higher or lower than the stimulation frequency for 4 Hz and 6 Hz, respectively. Thus, individual frequency adjustment was critical.

Video S1. Example video of one trial: action domain, expected outcome, 4 Hz.

Video S2. Example video of one trial: action domain, unexpected outcome, 4 Hz.

Video S3. Example video of one trial: action domain, expected outcome, 6 Hz.

Video S4. Example video of one trial: action domain, unexpected outcome, 6 Hz.

Video S5. Example video of one trial: number domain, expected outcome, 4 Hz.

Video S6. Example video of one trial: number domain, unexpected outcome, 4 Hz.

Video S7. Example video of one trial: number domain, expected outcome, 6 Hz.

Video S8. Example video of one trial: number domain, unexpected outcome, 6 Hz.

Preregistration.

The following preregistration is accessible via the following link:

http://aspredicted.org/blind.php?x=vr6cq2.

AsPredicted
Pre-Registration made easy

CONFIDENTIAL - FOR PEER-REVIEW ONLY

How infants encode unexpected events: a SSVEP study (#5189)

Created: 08/18/2017 06:55 AM (PT)
Shared: 10/26/2018 05:39 AM (PT)

This pre-registration is not yet public. This anonymized copy (without author names) was created by the author(s) to use during peer-review. A non-anonymized version (containing author names) will become publicly available only if an author makes it public. Until that happens the contents of this pre-registration are confidential.

1) Have any data been collected for this study already?
No, no data have been collected for this study yet

2) What's the main question being asked or hypothesis being tested in this study?
We assess infants' brain responses to unexpected events (violation of expectation response). Specifically, we will present sequences with a physically or socially expected or unexpected outcome (e.g. a ball falling through a table), while flickering these sequences at a theta (4 Hz) or alpha (6 Hz) frequency (rapid repetition paradigm). We will look at power differences in oscillatory responses at posterior electrodes (steady state visually evoked potentials; SSVEPs). We predict power differences between the unexpected and the expected outcome, possibly interacting with the driving frequency. That is, higher theta SSVEPs for the unexpected outcome, indicating an interaction with learning processes, but higher alpha SSVEPs for the expected outcome, indicating reduced attention.

3) Describe the key dependent variable(s) specifying how they will be measured.
The dependent variable is the SSVEP signal at posterior recording sites (focussing on electrodes O1, O2, Oz).

The SSVEP Signal will be preprocessed as follows:
- Artifact removal by ICA decomposition and visual inspection (Chaumon et al., 2015).
- Wavelet Analysis at the 2 SSVEP Frequencies (4 Hz, 6 Hz)
- Main Score: relative signal change in % (SSVEP Amplitude) for the outcome picture (3.0 – 4.5 after stimulus onset), relative to the preceding 1.5 sec (1.5 – 3 after stimulus onset). used as baseline.

4) How many and which conditions will participants be assigned to?
Design
- 4 Core Knowledge Domains (action, number, continuity, physics), with 4 stimulus variations (for each domain)
- 2 Outcomes (expected, unexpected)
- 2 Frequencies (alpha, theta)

Complete within-subjects design:
- 64 trials (4 Domains in 4 Variations, 2 Outcomes, 2 Frequencies)
- 4.5 seconds presentation of each trial: Initial situation (1.5 s), action/physical event (1.5 s), outcome (1.5 s).
- The presentation is blocked (4 stimuli per block), but may be paused after each trial.
- If the infant is still attentive, the whole presentation (ca. 10 minutes) will be repeated a second time

5) Specify exactly which analyses you will conduct to examine the main question/hypothesis.
For the extraction of the SSVEP signal from the EEG data see 3) Dependent Measure

ANOVA (3 within factors)
- 4 Core Knowledge Domänen (action, number, continuity, physics)
- 2 Outcomes (expected, unexpected)
- 2 Freq (alpha, theta)

Hypothesis:
1. Main Effect Outcome: Higher Amplitudes for unexpected opposed to expected outcomes
2. Outcome*Frequency interaction (possibly): Functional differentiation between Theta (higher for unexpected) and Alpha (higher [less supression] for expected).

6) Any secondary analyses?
3. Explorative: Different strength in effects for the 4 core knowledge domains? (effect of cognition or stimulus material?)
4. Explorative: temporal characteristics of the SSVEP Amplitude (similar to the temporal characteristics found in ERP Studies? Frequency Specific?)

7) How many observations will be collected or what will determine sample size? No need to justify decision, but be precise about exactly how the number will be determined.

Verify authenticity: http://aspredicted.org/blind.php?x=vr6cq2

Version of AsPredicted Questions: 1.05

Nine-month-old (9 months 0 days – 9 months 30 days) full-term born infants will be tested. The age was selected based on former studies indicating VOE responses for the core knowledge domains (e.g., Reid et al., 2009). Previous EEG studies used final samples between 10 and 50 infants (cf Hoehl & Wahl, 2012). As we apply a new EEG design and cannot yet estimate SSVEP effect sizes, we will test 35 infants, towards the upper end of the sample size range.

8) Anything else you would like to pre-register? (e.g., data exclusions, variables collected for exploratory purposes, unusual analyses planned?)
There are usually high drop out rates for infant EEG studies. Although we aim to collect 35 infants with good recording quality, the completition of this samplesize depents on the drop out and the availibility of infants in the Database. Thus it is possible that the final analysis includes some infants less

Verify authenticity:http://aspredicted.org/blind.php?x=vr6cq2

Version of AsPredicted Questions: 1.05
